# Turkish validity and reliability study of Self-Blame Attributions for Cancer Scale: A methodological study

**DOI:** 10.1017/S1478951526102065

**Published:** 2026-03-25

**Authors:** Gülcan Bahçecioğlu Turan, Nejla Demir, Zülfünaz Özer

**Affiliations:** 1 Department of Nursing, Faculty of Health Sciences, Firat University, Elazığ, Türkiye; 2 Department of Internal Medicine Nursing, Fırat University Institute of Health Sciences, Elazığ, Türkiye; 3 Department of Nursing, Faculty of Health Sciences, Istanbul Sabahattin Zaim University, İstanbul, Türkiye

**Keywords:** Nursing, patient, cancer, self-blame, validity, reliability

## Abstract

**Purpose:**

This study aimed to culturally adapt the Self-Blame Attributions for Cancer Scale (SBAC) into Turkish and evaluate its psychometric properties, including validity and reliability.

**Method:**

This methodological study enrolled 161 patients from both inpatient and outpatient oncology departments of a university hospital during a 1-year observation period (March 2024–March 2025). Participant data were obtained by using 2 instruments: a demographic questionnaire and the adapted Turkish version of “the SBAC.”

**Results:**

Confirmatory factor analysis revealed strong factor loadings ranging from 0.670 to 0.850, indicating good item reliability. Model fit statistics demonstrated excellent psychometric properties (χ^2^/*df* = 2.00; root mean square error of approximation = 0.079; Comparative Fit Index = 0.99; standardized root mean square residual = 0.042; Tucker–Lewis Index = 0.98; root mean square residual = 0.042). The scale showed high internal consistency, with a total Cronbach’s α of 0.93 and subscale α coefficients ranging from 0.85 to 0.90. The original 2-factor structure of the SBAC was supported.

**Conclusion:**

The study confirmed the bidimensional structure (11 items) of SBAC’s Turkish version with excellent validity and reliability indices, supporting its cultural and psychometric adequacy for Turkish samples.

## Introduction

Cancer is an increasing health problem both on a global and local scale. According to the World Health Organization (WHO) data for 2023, every year nearly 20 million new cases are reported and this number is expected to exceed 30 million by 2040 (World Health Organization (WHO) 2023). In Turkey, according to the data of the Ministry of Health, approximately 250,000 new cancer cases are diagnosed annually and these figures are increasing every year (T.C. Sağlık Bakanlığı Halk Sağlığı Genel Müdürlüğü Kanser Dairesi Başkanlığı 2020). Cancer has a profound impact not only on individuals’ physical, emotional, and psychological health (WHO 2020).

A diagnosis of cancer usually leads to various psychological reactions such as fear, anxiety, anger, helplessness, and guilt. Self-blame, which is one of these psychological reactions, occurs frequently, especially in the internal evaluations of the individual regarding the cause of the illness. Self-blame is associating a negative situation with one’s own fault, behavior, or personality traits (Janoff-Bulman 1979). This concept is addressed in 2 dimensions: Behavioural Self-Blame (BSB): It is the individuals’ belief that the event they have experienced is caused by the behaviors they have done or not done. For example, a patient may blame himself/herself with the thought “If I had eaten healthier, I would not have developed cancer.” Characterological Self-Blame (CSB): The individual believes that the event was caused by his/her own personality or an intrinsic defect. In such blaming, the individual associates an uncontrollable aspect of the event with his/her personal inadequacies: “I am a weak person, that’s why illnesses find me.” Self-blame reaction may negatively affect the psychological adjustment of individuals in serious disease processes such as cancer. In particular, CSB is associated with the development of feelings such as depression, anxiety, and hopelessness (Janoff-Bulman 1979; Bennett et al. 2005, 2013; Lebel et al. 2013; Jannati et al. 2020). Self-blame has a direct impact not only on the individual’s perception of the disease but also on his/her compliance with the treatment process and overall quality of life (Voth and Sirois 2009; Plaufcan et al. 2012; Eways et al. 2020).

Turkish cancer patients may embrace internal blame while also interpreting the illness through religious and fatalistic meanings, accepting the disease as “God’s decree” (Afsaroğlu et al. 2010; Yılmaz et al. 2017; Ahmadi et al. 2019). For example, in a comparative study of illness attributions, Turkish patients were significantly more likely to cite “God’s will” or “fate” as causal factors compared to German patients, who emphasized lifestyle and environmental risks (Thein et al. 2020). Such meanings can shape how patients engage with care: feeling personally responsible and/or viewing the illness as predetermined may lead some patients to rely more on family members, religious authorities, or other nonmedical sources, which can weaken physician-guided management and shared decision-making and may increase the risk of delayed or inappropriate treatment. Therefore, identifying the meanings patients attribute to the disease is important for improving treatment adherence and supporting coping with the illness (Afsaroğlu et al. 2010). In cultures with more collectivistic values, such as Turkey, tendencies toward self-blame in the context of illness may be shaped by cultural constructs such as fatalistic beliefs, concerns about burdening the family, feelings of modesty or shame, culturally embedded notions of guilt and religiously grounded explanatory frameworks (Ahmadi et al. 2019). Considering that self-blame may vary across cultures and that each culture holds its own belief systems, perceptions, and interpretations regarding health and illness, the need for culturally specific assessment tools becomes evident. Understanding patients’ attitudes toward self-blame is essential for the development of effective cancer education and psychoeducational programs (Yılmaz et al. 2017). Scientific evaluation of these internal blame attributions is very important in cancer patients. “The Self-Blame Attributions for Cancer Scale (SBAC)” developed by Eways et al. (2020) is one of the first instruments developed for this purpose (Eways et al. 2020). This scale allows individuals to self-evaluate their perceptions of their illness and their level of self-blame (Bennett et al. 2013). In this context, it is of great importance for both academic research and psychosocial support services to better understand why the concept of self-blame is important for cancer patients and to develop culturally adapted measurement tools that can be used in clinical assessment processes. In addition, since SBAC contains 11 items, it shows excellent practical feasibility due to its short administration time. There is a lack of measurements in Turkey used to assess cancer patients’ self-blame attributions. Therefore, the aim of present study was to adapt SBAC developed by Eways et al. (2020) into Turkish and to test its validity and reliability. Results of the present study will help health professionals to evaluate the self-blame status of cancer patients and to develop evidence-based interventions for this issue. It will also contribute significantly to the studies evaluating the self-blame status of cancer patients for researchers.

## Methods

### Design

The present study is a methodological research.

### Participants and sample

The research was carried out in oncology inpatient and outpatient departments of a university-affiliated medical center during a 12-month study period (March 2024–March 2025). Population included patients diagnosed with cancer who were admitted to the hospital during this process, while the sample was selected from individuals who met the specified criteria. Being ≥18 years old, absence of psychiatric disorders, and communication ability were among the inclusion criteria. A total of 161 patients who met these conditions and gave consent for participation were included in the study. Literature emphasizes that at least 5–10 times as many participants as the scale items should be reached in scale development and adaptation studies (Seçer 2020; DeVellis and Thorpe 2021). Considering that the original scale consists of 11 items, between 50 and 110 participants were determined as the required sample size. However, 161 participants were included in the study.

## Data collection instruments

### “Descriptive Information Form” and SBAC were used in the study as data collection instrument

**Descriptive Information Form**: Patient demographics included age, sex, education, marital status, employment, diagnosis year, and chronic disease comorbidity

**SBAC**: It was developed by Eways et al. in 2020 to evaluate cancer patients’ self-blame for cancer. The 5-point Likert scale has 11 items scored as “Not at all (0), A little (1), Somewhat (2), A lot (3), Completely (4).” It has 2 subdimensions. These subdimensions are “BSB and CSB.” BSB subdimension consists of items 1–6. Scoring is between 0 and 24. CSB subdimension consists of items 7–11. Scoring is between 0 and 20. The total scale is scored between 0 and 44. Higher scores show higher self-blame related to the disease. There are no reversely coded items. In the original scale, Cronbach’s alpha values vary between 0.76 and 0.91 (Eways et al. 2020).

## Adaptation process

**Language and Content Validity**: Five stages (“forward translation, expert opinion, backward translation, pretest, and final version”) were followed to conduct the study by using a systematic approach complying with the WHO translation guidelines (World Health Organization (WHO) 2017). In this context, the SBAC was translated into Turkish by 2 linguists. The translated scale was subsequently submitted to a panel of 10 content experts specializing in internal medicine nursing for validation. A panel of experts evaluated content validity using “the Content Validity Index (CVI)” methodology. Following the Davis technique (Çapık et al., 2018), experts independently rated each item on a 4-point Likert scale assessing both (1) linguistic equivalence between original and translation versions, and (2) conceptual clarity of each statement. This systematic approach enabled quantitative evaluation of item appropriateness while maintaining rigorous standards for cross-cultural adaptation. Experts evaluated each item in 4-point Likert scale, where 1 indicated inappropriate (requiring rejection), 2 denoted somewhat appropriate (needing major revision), 3 represented quite appropriate (requiring minor modifications), and 4 signified fully appropriate (no changes needed). CVI was derived by calculating the rate of experts who rated an item as 3 or 4 (i.e., appropriate with/without minor revisions) relative to the total number of evaluators. Relevant corrections were made to the items in the scale using the feedback from the experts. Following the corrections, an individual not involved in the first translation translated the scale back into English. The resulting version was sent to the authors of the original scale and was approved by the authors. The scale was then piloted with 30 cancer patients and it was tried to determine whether it was applicable for the patients and the scale form was found to be understandable. The study did not include these data.

**Construct Validity**: In evaluating scale structures, factor analysis is conducted to test construct validity. “Confirmatory factor analysis (CFA)” evaluates each item forming the factors to find out whether correlation with the factor is sufficient. CFA is a method of finding evidence of validity that can be used especially in the adaptation of scales (Karaman 2023). “Kaiser–Meyer–Olkin (KMO)” sampling adequacy measure and Bartlett’s Sphericity test are taken into consideration to evaluate the suitability of the data for factor analysis in a study. Before applying factor analysis, KMO test was conducted to verify sample adequacy and Bartlett’s test of sphericity was conducted to confirm the suitability of the data for factor analysis (Seçer 2020; Shrestha 2020). KMO value of >0.60 and statistically significant results from Bartlett’s test of sphericity indicates that the sample is sufficient and the data set is suitable for factor analysis (Seçer 2020). Goodness-of-fit indices were analyzed to determine the most appropriate structure for the scale in the application of CFA (Bae 2017; Woo 2017). General fit coefficients (Relative Chi Square Index [CMIN/*df*], root mean square error of approximation [RMSEA], Comparative Fit Index [CFI], Tucker–Lewis Index [TLI], standardized root mean square residual [SRMR], root mean square residual [RMR]) and factor loadings were calculated in CFA for different scale dimensions (Karaman 2023). If the fit indices of the scale are to be considered satisfactory, the CMIN/*df* value should be less than 5, the RMSEA value should be less than 0.08, the CFI and TLI values should be more than 0.90, and the SRMR and RMR values should be less than 0.08 (Byrne 2013; Bae 2017; Woo 2017; Seçer 2020). Factor analysis results should show >0.30 factor loadings for the items. However, if the results are between 0.30 and 0.59, they are at medium level and if they are 0.60 and above, they are reported to be at high level (Karaman 2023). Heterotrait–Monotrait ratio (HTMT) was computed to evaluate discriminant validity as the ratio of the mean of the absolute correlations across indicators of different constructs (heterotrait–heteromethod) to the geometric mean of the mean absolute correlations among indicators within the same construct (monotrait–heteromethod) (Henseler et al. 2015). HTMT was calculated using Pearson correlations in SPSS.

**Convergent Validity:** Convergent validity was determined with “average variance extracted (AVE)” and “Composite Reliability (CR)” values (Alarcón et al. 2015). AVE value is expected to >0.50 and CR value is expected to be >0.80 (Netemeyer 2003). Besides, the conditions of CR > AVE and AVE > 0.50 were taken into consideration to ensure convergent validity (Yaşlıoğlu 2017).

**Reliability Analyses**: In order to determine the reliability of the Turkish version of the SBAC, internal consistency coefficients, item-total correlations, and test–retest correlations were performed. Acceptable levels were determined as Cronbach’s alpha coefficient ≥0.70 for the whole scale, >0.30 for the item-total correlations, and >0.70 for the correlations between the measurements made at 2-week intervals for the test–retest application (Creswell and Creswell 2017; Seçer 2020). By determining whether or not the items measure the same concepts and whether or not the items are truly connected to what is being measured, the internal consistency coefficient evaluates the items. The internal consistency coefficient of a measurement instrument should be as close as possible to 1 in order to be considered an indicator of adequate reliability. Internal consistency of the scale was evaluated with Cronbach’s alpha coefficient and McDonald Omega reliability analysis. An internal consistency coefficient >0.70 is considered sufficient for the scale to be considered reliable (Pallant 2020; Seçer 2020). Item-total correlation coefficient is the indicator frequently used in item selection analyses to understand the degree to which scale items are related to the whole scale. A high correlation coefficient of an item indicates that the measured item has a high correlation with the theoretical structure of that item and that the item is effectively sufficient to measure the desired behavior. Item-total correlation coefficient of measurements obtained from a scale should be >0.30 (Tabachnick and Fidell 2015; Kartal and Bardakçı 2018). Test–retest measurements evaluate the consistency of an instrument over time and are one of the most frequently used reliability analyses. In calculating how consistent a measurement instrument is over time, reliability is considered to be high as the correlation coefficient approaches 1. Correlation coefficients between the test–retest scores obtained in determining that the measurements obtained from the scale are invariant over time should show a high level of positive correlation and this score should be at least 0.70 for acceptability (Tavşancıl, 2014). In addition, “the intraclass correlation coefficient (ICC)” was used in test–retest measurements. The ICC value varies between 0 and 1; a value approaching 1 indicates that the measurements are highly consistent (Koo and Li 2016). To assess test–retest reliability, the scale was readministered to a subsample of 40 patients drawn from the main sample. The retest took place after an average of 18.6 ± 3.9 days (range: 14–28 days) following the initial administration. This 2- to 4-week interval was selected to minimize potential learning effects while reducing the likelihood of substantial changes in participants’ clinical status or treatment regimen. The retest subsample was formed using a consecutive convenience approach, including participants who could be reached in the order of their initial participation and who agreed to complete the second assessment.

## Statistical analysis

All analyses were conducted using SPSS 27.0 and LISREL software. Descriptive statistics (frequencies, percentages, means, and standard deviations) characterized the sample demographics. To assess construct validity, we performed CFA in LISREL, examining multiple fit indices. Scale reliability was evaluated through 3 methods: (1) item-total correlations, (2) Cronbach’s alpha coefficients, and (3) McDonald’s omega coefficients. Additionally, test–retest reliability analysis determined the scale’s stability over time. A significance threshold of *p* < 0.05 was applied for all tests.

## Ethical considerations

This study received formal approval from the University Ethics Committee (Approval No: XXX) and institutional authorization from the participating hospital. Written permission for cross-cultural adaptation of the SBAC scale was secured via email correspondence with the author of the original scale. The ethical norms that are mentioned in the Declaration of Helsinki were adhered to in a stringent manner throughout the research. Immediately after receiving a comprehensive description of the study’s objectives and procedures, each participant gave their verbal agreement after being informed.

## Results

The participants were between the ages of 18 and 80 and mean age was found as 55.09 ± 13.80 years. Of the participants, 50.3% were male, 62.1% were married, 37.9% were primary school graduates, 75.8% were not employed, 24.8% had lung cancer, 43.3% had stage III disease, 55.9% received chemotherapy treatment, and 55.9% had no other chronic disease. It was also found that the range of diagnosis period of the participants was between 1 and 4 years and the mean diagnosis was 1.55 ± 0.84 (Table 1).

**Table 1.** Descriptive characteristics (n= 161)

Characteristics	Number (*n* = 161)	%
**Gender**		
Female	80	49.7
Male	81	50.3
**Marital status**		
Married	100	62.1
Single	61	37.9
**Educational status**		
Literate	5	3.1
Primary Education	61	37.9
Secondary Education	19	11.8
High School	47	29.2
Undergraduate and above	29	18.0
**Employment status**		
Yes	39	24.2
No	122	75.8
**Disease**		
Breast	15	9.3
Thyroid	5	3.1
Lung	40	24.8
Gastrointestinal	27	16.8
Gynecological	18	11.2
Lymphoma	6	3.7
Multiple myeloma	7	4.3
Prostate	13	8.1
Rectum	18	11.2
Brain	7	4.3
Kidney	2	1.2
Skin	3	1.9
**Stage of the disease**		
I	12	7.5
II	67	41.6
III	70	43.5
IV	12	7.5
**Treatment received**		
Chemotherapy	90	55.9
Radiotherapy	7	4.3
Chemotherapy–Radiotherapy	41	41.0
Chemotherapy-Surgery	23	14.3
**Presence of other chronic diseases**		
Yes	71	44.1
No	90	55.9
	Mean ± SD	Min-Max
**Age (years)**	55.09 ± 13.80	18−80
**Duration of diagnosis**	1.55 ± 0.84	1−4

## Findings related to validity

### Content validity

In the current investigation, the range of values for the item-based CVI was between 0.90 and 1.00, and the scale-based CVI was 0.96.

## Construct validity

Prior to the implementation of construct validity, the KMO and Barlett’s Sphericity Tests were carried out in order to determine whether or not the sample size and data set were suitable for analysis. KMO value was 0.934 and Barlett’s Sphericity Test was significant (χ^2^ = 1143.738; *p* < 0.001). Validity of the 11-item 2 subdimensional structure was examined with CFA analysis, resulting in the factor loadings to vary between 0.670 and 0.850 (Table 2).

**Table 2.** Mean, item correlation coefficient, and CFA factor load results

				Factor load value	
Scale items		Mean ± SD	Corrected item-total correlations	F1	F2
Item1	*How much do you blame yourself for past behaviors that may have caused your cancer?*	1.95 ± 0.98	0.812	0.850	
Item2	*To what extent do you accept fault for behaviors that may have caused your cancer?*	1.81 ± 0.97	0.765	0.790	
Item3	*How much do you think your past behaviors contributed to your cancer?*	1.95 ± 0.85	0.697	0.700	
Item4	*To what extent do you believe that a change in your behavior could have prevented your cancer?*	1.65 ± 1.02	0.730	0.740	
Item5	*To what extent do you feel accountable when thinking about past behaviors that may have caused your cancer?”*	1.79 ± 1.02	0.729	0.780	
Item6	*When discussing possible causes of your cancer with important people in your life, to what extent have you blamed your past behavior?*	1.88 ± 0.96	0.748	0.810	
Item7	*How much do you blame the type of person you are for your cancer?*	1.55 ± 1.16	0.802		0.850
Item8	*To what extent do you believe that a change in the type of person you are could have prevented your cancer?*	1.82 ± 1.05	0.630		0.680
Item9	*How much do you blame your personality for your cancer?*	1.54 ± 1.13	0.706		0.750
Item10	*How much do you blame yourself for being the type of person who has bad things, like cancer, happen to them?*	1.72 ± 1.05	0.664		0.700
Item11	*When discussing possible causes of your cancer with important people in your life, to what extent have you blamed your personality?*	1.86 ± 0.81	0.653		0.670

Factor 1 = behavioral self-blame; Factor 2 = characterological self-blame.

## Confirmatory factor analysis

**Figure 1.**
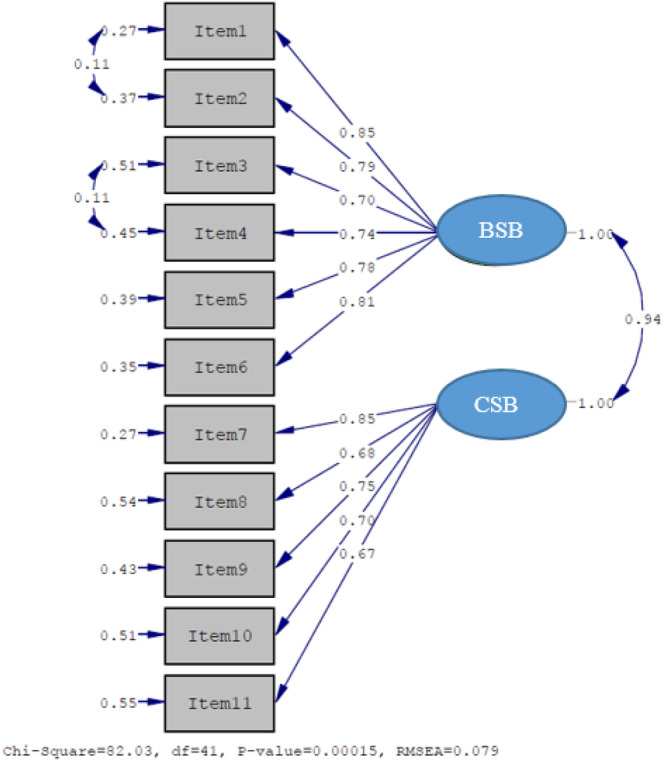
PATH diagram regarding the factor structure of the scale.

The 1-factor model showed poorer fit, χ^2^(44) = 118.20, *p* < 0.001, χ^2^/*df* = 2.69, RMSEA = 0.11, CFI = 0.97, TLI = 0.97, SRMR = 0.047, and RMR = 0.048. In contrast, the hypothesized 2-factor model demonstrated superior fit, χ^2^(41) = 82.03, *p* < 0.001, χ^2^/*df* = 2.00, RMSEA = 0.079, CFI = 0.99, TLI = 0.98, SRMR = 0.042, and RMR = 0.042, supporting retention of the 2-factor structure (Table 3). Figure 1 shows the PATH diagram of the CFA.

BSB =behavioral self-blame; CSB = characterological self-blame.

**Table 3.** Confirmatory factor analysis results

Model	χ^2^/*df*	RMSEA	CFI	SRMR	RMR	TLI
**One-factor**	2.69	0.11	0.97	0.047	0.048	0.97
**Two-factor**	2.00	0.079	0.99	0.042	0.042	0.98
**Appropriate**	*<2*	*<0.05*	*>0.95*	*<0.05*	*<0.05*	*>0.95*
**Acceptable**	*<5*	*<0.08*	*>0.90*	*<0.08*	*<0.08*	*>0.90*

CFI = Comparative Fit Index; RMSEA = root mean square error of approximation; RMR = root mean square residual; SRMR = standardized root mean square residual; TLI = Tucker–Lewis Index.

## Convergent validity

Convergent validity of the factors was examined with AVE values, which were found as F1 = 0.608 and F2 = 0.537, respectively. CR values were also calculated, which were found as F1 = 0.903 and F2 = 0.852, respectively (Table 4).

**Table 4.** Correlation values, reliability values

Scale and subdimensions	F1	F2	SBAC total	AVE	CR	α	Ω
**F1**	1			0.608	0.903	0.907	0.907
**F2**	*r* = 0.826**	1		0.537	0.852	0.850	0.857
SBAC total	*r* = 0.962**	*r* = 0.949**	1	-	-	0.933	0.934

F: Factor; SBAC = Self-Blame Attributions for Cancer Scale; Factor 1 = Behavioural self-blame; factor 2 = characterological self-blame.

** Correlation is significant at the 0.01 level (2-tailed).

## Findings related to reliability

Cronbach’s alpha coefficients were 0.907 for the “F1” subdimension, 0.850 for the “F2,” and 0.933 for the total scale. Omega reliability values were 0.907 for “F1,” 0.857 for “F2,” and 0.934 for the whole scale (Table 4). Item-total correlation coefficients were between 0.630 and 0.812 (Table 2).

A statistically significant correlation (*p* < 0.01) was found between the test–retest readings, with *r* = 0.943 for the SBAC total, *r* = 0.875 for the “F1” subdimension, and *r* = 0.834 for the “F2” subdimension. Based on the findings of the test–retest measurements, there was no statistically significant difference (Table 5) (*p* > 0.05). There was a range of 0.909–0.970 for the ICC values for test–retest reliability.

**Table 5.** Test–retest results and score means (*n* = 40)

	Scale score means		Analysis results				
Scale and subdimensions	First implementation (X ± SD)	Second implementation X ± SD	*r*	*p*	*t*	*p*	ICC
**F1**	10.46 ± 5.94	10.00 ± 5.83	0.875**	0.000	0.979	0.334	0.933
**F2**	8.17 ± 4.44	8.46 ± 4.46	0.834**	0.000	−0.671	0.506	0.909
SBAC total	18.64 ± 9.71	18.46 ± 9.19	0.943**	0.000	0.346	0.732	0.970

Factor 1 =behavioral self-blame; Factor 2 = characterological self-blame.

** Correlation is significant at the 0.01 level (2-tailed); F: Factor; *p* < 0.05; *r* = Pearson correlation coefficient; *t: t* = paired sample *t*-test; ICC = intraclass correlation coefficient; SBAC = Self-Blame Attributions for Cancer Scale.

## Discussion

In this study, the SBAC was adapted into Turkish and demonstrated overall satisfactory psychometric performance. The hypothesized 2-factor structure (BSB and CSB) was supported, internal consistency and temporal stability were high, and convergent validity was acceptable (Seçer 2020).

Regarding construct validity, the suitability of the dataset for factor analysis was confirmed with a KMO value of 0.93 (excellent) (Çokluk et al. 2014) and Bartlett’s test of sphericity (χ^2^ = 1143.738, *p* < 0.001). CFA verified that the original 2-dimensional structure BSB and CSB were preserved in the Turkish sample, with factor loadings ranging from 0.670 to 0.850. Overall goodness-of-fit indices were excellent (Seçer 2020). Factor loadings were between 0.670 and 0.850 in the present study. Similar to the original version, the CFA factor loadings were >0.30, indicating that the 11-item 2-dimensional measurement instrument (BSB, CSB was valid.

For convergent validity, CR values (F1 = 0.903; F2 = 0.852) were higher than the respective AVE values (F1 = 0.608; F2 = 0.537), and all AVE values exceeded 0.50 (Yaşlıoğlu 2017). CFA revealed a high correlation between F1 and F2 (*r* = 0.826, *p* < 0.001). According to the Fornell–Larcker criterion, discriminant validity was not fully achieved; the shared variance between the 2 factors (0.826^2^ = 0.682) exceeded the AVE values of both factors (F1 = 0.608; F2 = 0.537) (Table 4). This finding indicates that Turkish cancer patients do not perceive BSB and CSB dimensions as sufficiently distinct. In the Turkish cultural and religious context, self-blame is often experienced as a holistic feeling of “guilt/sinfulness/shame.” Concrete behaviors such as smoking, unhealthy diet, or stressful lifestyle are frequently intertwined with characterological interpretations such as “I am a weak-willed person,” “I am lazy,” or “I have a self-destructive personality” (Ahmadi et al. 2019; Zhao et al. 2020). Consequently, patients do not draw as sharp a distinction between “I could have changed my behavior” and “This is who I am” as observed in Western samples; the 2 dimensions tend to merge into a single general self-blame experience. In the present study, the HTMT ratio between BSB and CSB was 0.892. Although below the commonly used upper bound of 0.90, this value is notably high and suggests substantial overlap between the 2 dimensions (Diamantopoulos et al. 2012; Roemer et al. 2021). Nevertheless, the 2-factor model fit the data better than a 1-factor alternative, indicating that the subscales capture meaningfully distinct facets despite their close association. Accordingly, the 2 subscales were retained and were not merged into a single factor solely on the basis of the original structure, but because they represent theoretically distinct components of self-blame and the 2-factor model showed superior fit relative to the 1-factor model. Given the strong association, reporting both subscale scores and a total score may be pragmatic depending on the intended use.

Reliability analyses revealed Cronbach’s alpha coefficients of 0.93 for the total scale and 0.90 (BSB) and 0.85 (CSB) for the subscales; Omega coefficients also exceeded 0.85. Item-total correlations ranged from 0.664 to 0.812, with all items meeting acceptance criteria (Tabachnick and Fidell 2015; Kartal and Bardakçı 2018). Test–retest correlations were above 0.83, and the ICC was >0.90, confirming that the scale is consistent over time and exhibits excellent reliability (DeVellis and Thorpe 2021). Overall, the test–retest findings support the temporal stability of the scale.

One of the notable findings of the present study is that the item scores of the scale were higher than those reported in the original validation study by Eways et al. (2020). Although fatalistic beliefs and the notion of illness as “God’s decree” are deeply rooted in Turkish society, these beliefs do not entirely eliminate self-blame; rather, they may coexist with intensified feelings of guilt, shame, and sinfulness in some patients (Ahmadi et al. 2019) . A subset of Turkish cancer patients interprets their illness as “punishment for past sins” or “a divine punishment,” which in turn heightens both CSB and BSB. In contemporary Turkey, particularly among urban, educated, and younger patients, traditional fatalistic explanations and modern notions of personal responsibility tend to coexist. Behaviors such as smoking, stress, and poor diet can be simultaneously labeled as both “fate” and “my own fault,” creating a dual explanatory framework. This overlap contributes to elevated self-blame scores (Afsaroğlu et al. 2010; Ahmadi et al. 2019). This pattern suggests that the higher mean scores observed for the SBAC in Turkey are not indicative of a failure of the instrument but rather a cultural reflection demonstrating that the concept of self-blame operates in a more complex and multifaceted manner within the Turkish cultural context compared to Western settings.

Practical implications for supportive and palliative care follow directly from these findings. First, routine psychosocial screening in oncology can incorporate brief questions about self-blame, guilt, and shame at key time points (diagnosis, major treatment transitions, recurrence/progression, and initiation of palliative care) to identify patients at risk for distress, avoidance, or disengagement from care. Second, elevated self-blame should activate clear referral pathways to psycho-oncology or clinical psychology (for shame-informed counseling, cognitive restructuring of self-blaming beliefs, and self-compassion-focused strategies), psychiatry when depressive or anxiety symptoms are prominent, and social work for practical stressors that can intensify self-criticism. Third, clinicians can use non-stigmatizing communication that distinguishes “responsibility” from “fault” and frames risk behaviors as modifiable health factors rather than moral failures; brief normalization and validation can reduce shame-related avoidance. Finally, when self-blame is tied to spiritual meanings (e.g., sinfulness or punishment), collaboration with trained spiritual care providers (where available) and family-inclusive conversations may help address moral distress without reinforcing blame. Integrating these steps into a simple workflow screen, provide brief support, refer, and follow-up may strengthen coping and engagement across the disease trajectory.

## Limitations of the study

An important limitation of the study is the fact that it was conducted in a single center. The results obtained are valid only for the patients who participated in the study and cannot be generalized to the entire patient population. In addition, the study data are based on patients’ self-reports. Responses given depending on the subjective perceptions of individuals should be taken into consideration as a factor that may affect the reliability of the data. Furthermore, while cultural factors were considered in adaptation, the sample’s homogeneity may not fully capture regional variations within Türkiye.

## Conclusion

The Turkish version of SBAC is a valid, reliable, and practical tool for assessing self-blame in cancer patients. Its brevity and ease of administration support routine use in clinical practice, enabling healthcare professionals to identify patients at risk for maladaptive self-blame and provide targeted supportive interventions. By explicitly considering cultural factors such as family dynamics, societal beliefs, and language, the scale can inform culturally sensitive care strategies and enhance patient outcomes. Future research should examine the applicability of SBAC across diverse regions and patient populations to confirm generalizability and explore potential cultural variations in self-blame patterns. Integrating this tool into clinical practice and research can improve understanding of patients’ psychological experiences, guide interventions, and ultimately contribute to more effective palliative and supportive care.
